# Clinical and Radiographic Evaluation of Soft Tissue and Bone Status in Immediate Loaded Implants Placed Following Their Extraction in the Maxillary Anterior Region

**DOI:** 10.7759/cureus.68613

**Published:** 2024-09-04

**Authors:** Thenmozhi R, Sriraam KG, Charumathi S, Vikraman Baskara Pandian

**Affiliations:** 1 Oral and Maxillofacial Surgery, Ragas Dental College and Hospital, Chennai, IND

**Keywords:** 3d models, rapid prototyping, patient specific, customised abutments, maxillary anterior region, immediate loaded implants, esthetics, soft tissue preservation, emergence profile, bone status

## Abstract

Introduction: In the maxillary anterior region, teeth extraction leads to significant soft and hard tissue changes. Immediate implant placement following extraction aims to reduce bone loss and overall treatment time. However, it may result in adverse soft tissue changes impacting esthetics. This study evaluates the clinical and radiographic outcomes of immediately loaded implants in the maxillary anterior region, focusing on soft tissue preservation and bone status.

Materials and methods: This study, conducted from April 2022 to August 2024 at the Department of Oral and Maxillofacial Surgery, Ragas Dental College and Hospital, included 10 immediately loaded implants in seven patients. Following atraumatic extraction, implants were placed and loaded with functional provisional crowns fabricated using three-dimensional (3D) rapid prototyping models. Parameters such as crestal bone loss, buccal and palatal bone width, and interdental papilla thickness were evaluated preoperatively and postoperatively using radiographs and clinical assessments.

Results: The study found significant crestal bone loss at both mesial and distal sites over time, with the greatest loss observed at the three-month follow-up. Buccal and palatal bone width showed no significant differences preoperatively and postoperatively. Interdental papilla thickness and overall pink esthetic scores also showed no significant differences between preoperative and postoperative evaluations.

Conclusion: Immediate implant placement in the maxillary anterior region, using 3D rapid prototyping for custom splint fabrication, demonstrated effective preservation of soft tissue profile and bone architecture. This approach provides functional and esthetic benefits, although careful monitoring of crestal bone loss is necessary.

## Introduction

In the maxillary anterior region, teeth are extracted due to various reasons. Both soft tissue and hard tissue undergo qualitative and quantitative changes in the post-extraction phase [1,2]. Dental implants have proven to be an efficient option for replacing teeth. When implants are placed in insufficient bone, it may lead to adverse angulation, mechanical overload, and aesthetic problems resulting in implant failure. Thus, either alveolar ridge preservation or bone augmentation procedures were carried out prior to implant placement [3].

In order to reduce the bone loss and overall treatment time, implants were placed immediately following the atraumatic extraction of the tooth [4,5]. However, it presented with soft tissue changes around the implant-placed region, including papillary recession, resulting in the formation of a black triangle after fixing the prosthesis, loss of the width of attached gingiva, and alteration of gingival contour affecting the emergence profile during the final prosthesis placement. For a tooth in the maxillary anterior region, the emergence profile is of utmost importance for esthetics [6].

In order to maintain the bone architecture along with the soft tissue profile in the maxillary esthetic zone, immediate prosthetic restoration on immediately placed implants following atraumatic extraction will be of better choice [7,8].

The aim of this study is to clinically and radiographically assess the outcomes of immediate implant placement and functional provisional loading following tooth extraction in the maxillary anterior region. The provisional crown is created using an implant with a prepared abutment and is placed in a 3D rapid prototype (RP) model of the anterior maxillary region, which is manufactured from cone beam computed tomography (CBCT)/conventional computed tomography (CT) scans.

The objectives of this study are to preserve the soft tissue around the implant and maintain the gingival contour and the width of the attached gingiva. Additionally, the study aims to reproduce the emergence profile in the final prosthesis, ensure the retention of interdental papillae, and minimize marginal bone loss. Furthermore, the study seeks to prepare a provisional prosthesis (crown) preoperatively using a 3D-printed RP model of the anterior maxillary region.

## Materials and methods

This study is a clinical trial focused on evaluating the outcomes of immediate implant placement and functional provisional loading following tooth extraction in the maxillary anterior region. It involves a prospective design where the researchers follow a cohort of patients over time to assess the effectiveness of the procedure and its impact on various clinical and radiographic parameters. The study incorporates both preoperative and postoperative assessments, including clinical evaluations (e.g., pink esthetic score (PES)) and radiographic measurements (e.g., bone levels using intraoral periapical radiograph (IOPA) and CBCT).

The study was conducted between the time period of April 2022 and August 2024 in the Department of Oral and Maxillofacial Surgery, Ragas Dental College and Hospital, and ethical clearance was obtained from the Institutional Review Board (EC/NEW/INST/2023/4006). The clinical trial has been successfully registered with the reference number REF/2024/04/081891.

A total of 10 immediately loaded implants placed in seven patients were included in this study. The patients were selected according to inclusion and exclusion criteria, and informed consent was obtained for the same. In the maxillary esthetic region (teeth number 21), following the atraumatic extraction of a tooth, implants were placed immediately and loaded with a functional provisional crown, prepared preoperatively in the 3D-printed RP model of the anterior maxillary region. The final prosthesis was provided after three months.

The tooth that cannot be saved by any other means of treatment in the anterior region of the maxilla, adequate interocclusal space with dentition in the opposing arch, intact buccal bone before and after extraction, age >18 years, good state of systemic and oral health, healthy periodontal conditions of adjacent teeth, and subjects who are willing to participate in the follow-up study were included in the study.

Severe periodontitis with bone loss, tooth with large apical pathology, buccal plate deficiency before or after extraction, apical pathology in adjacent teeth, pregnancy, heavy smoking (>10 cigarettes per day), contradictory systemic diseases, and systemic bone diseases were excluded from the study.

Preoperative and postoperative evaluation of parameters were as follows: In the IOPA, crestal bone loss was evaluated on both mesial and distal aspects at immediate postop, two weeks, four weeks, six weeks, and eight weeks of follow-up. In CBCT, buccal and palatal bone widths were evaluated. Clinically, PESs were evaluated. In the study models, the thickness of the interdental papilla was evaluated on both mesial and distal aspects.

The PES is utilized in this study to objectively assess the soft tissue esthetics around the implant site, particularly in the maxillary anterior region where esthetics are critical. Since the study involves immediate implant placement and functional provisional loading, maintaining the natural appearance of the soft tissues, including the gingival contour, interdental papilla, and overall soft tissue harmony with adjacent teeth, is crucial. The PES provides a standardized method to evaluate these esthetic outcomes by analyzing parameters such as the presence and quality of the mesial and distal papillae, soft tissue level, contour, color, and texture. This assessment helps ensure that the immediate loading of implants achieves not only functional success but also meets high esthetic standards, which is especially important in the anterior maxilla where the visual outcome is a key concern for both clinicians and patients.

Preoperatively, digital imaging and communications in medicine (DICOM) data of CBCT/CT scans were obtained. Using “Mimics” software, a simulation of the extracted socket was done for the tooth of interest in the maxilla. 3D computer-aided designing (CAD) models of maxilla with simulated extracted socket and mandible were created. 3D CAD models were converted to binary stereolithographic model (STL) file formats. 3D RP models were printed by the additive manufacturing technique in Primaeam Solutions Private Limited (Chennai, India).

The 3D RP model of the maxilla without the tooth of interest (simulated extraction socket) was obtained. Drilling was done in the apical portion of the simulated extracted socket. The selected root form implant was positioned in the simulated socket at least 3 mm beyond the apex for anchorage and 1 mm below the marginal bone level. The implant is palatally placed to preserve the intact buccal bone and to maintain jumping distance.

The "jumping distance", which refers to the gap between the implant surface and the surrounding bone within the socket, was carefully maintained. This distance is critical in promoting bone regeneration around the implant.

**Figure 1:**
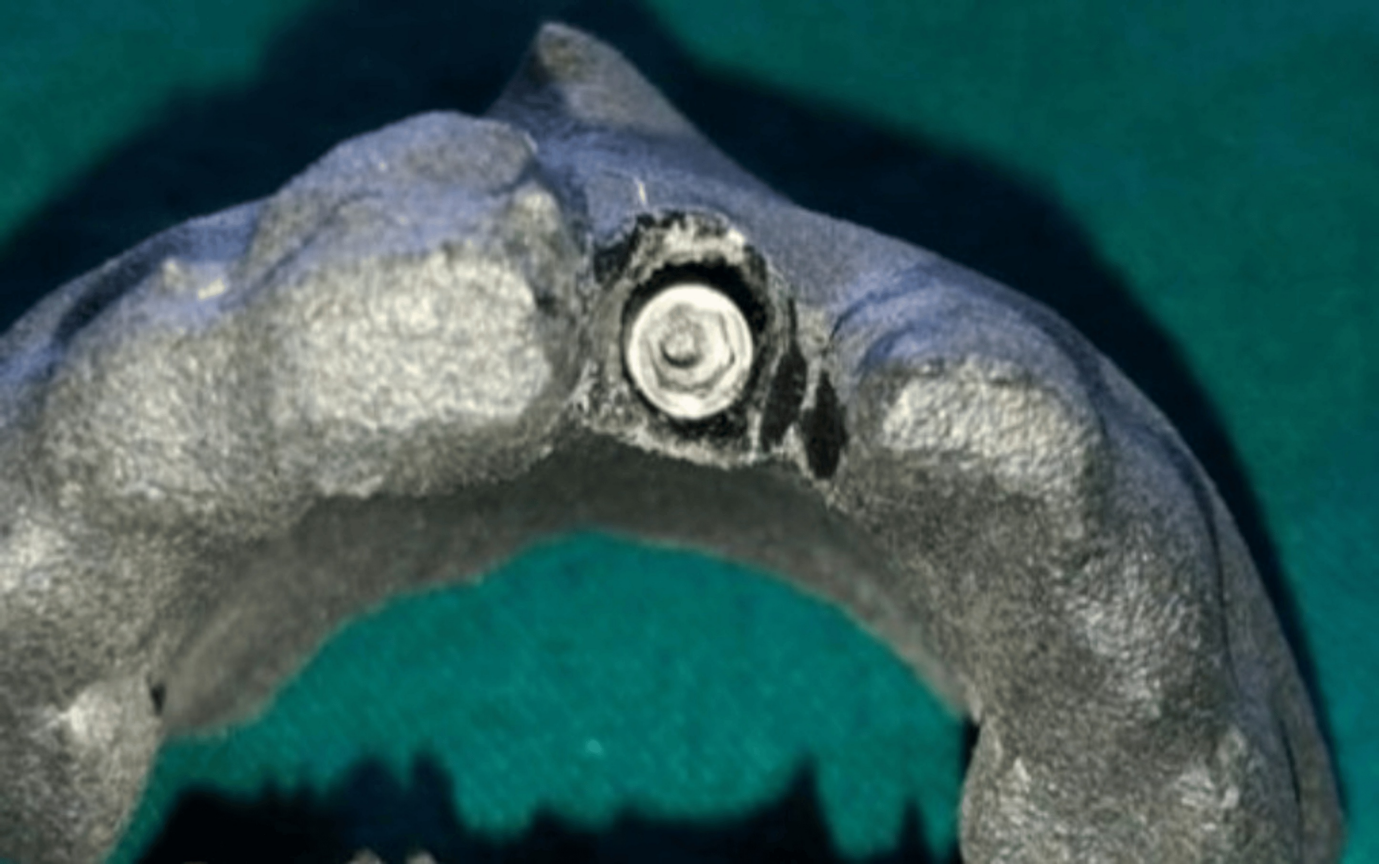
Implant placement in 3D RP models RP: Rapid prototype

Regarding the acceptable jumping distance without the need for grafting, studies suggest that gaps of up to 2 mm between the implant and the surrounding bone can often heal naturally without the necessity of bone grafting, as the body's natural healing processes are typically sufficient to fill this space with new bone. However, gaps larger than 2 mm may require grafting to ensure optimal bone integration and implant stability (Figure 1).

**Figure 2:**
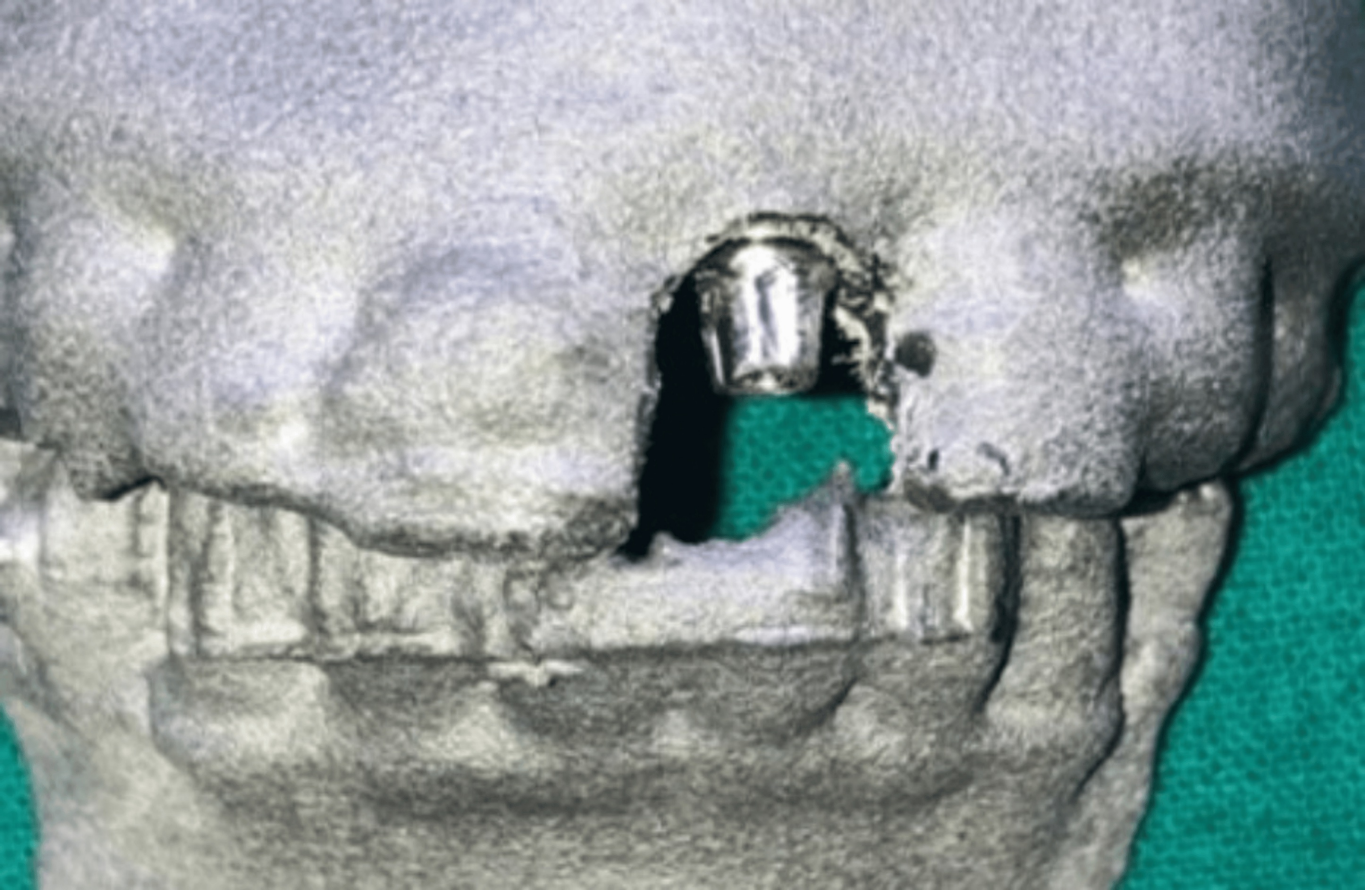
Prepared abutment in 3D RP models RP: Rapid prototype

The abutment was placed and trimmed accordingly with an opposing 3D RP model of the mandible. Orientation marking was done on the prepared abutment for accurate transfer to the patient (Figure 2).

**Figure 3:**
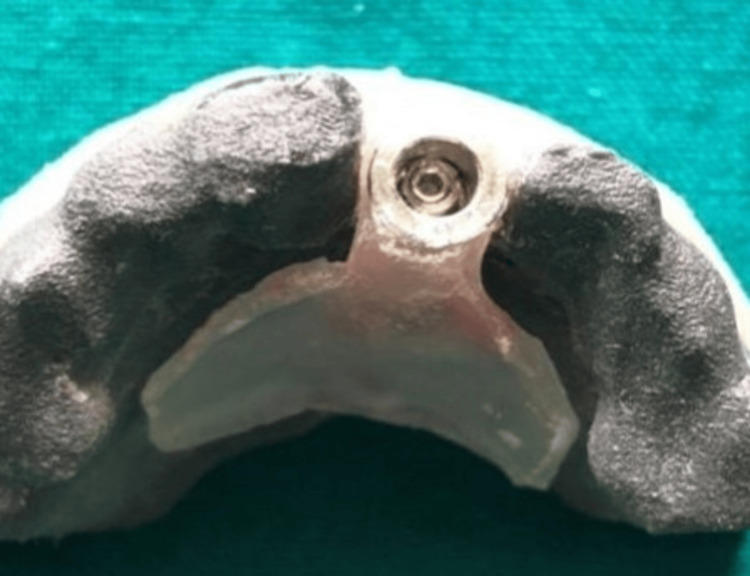
Surgical stent fabricated in 3D RP models RP: Rapid prototype

The 3D RP model of the maxilla was replicated with the prepared abutment with the Plaster of Paris (POP) cast. A provisional acrylic crown was fabricated in the prepared POP cast. A surgical stent in the 3D RP model with the prepared abutment was prepared for the appropriate transfer of implant position (Figure 3).

**Figure 4:**
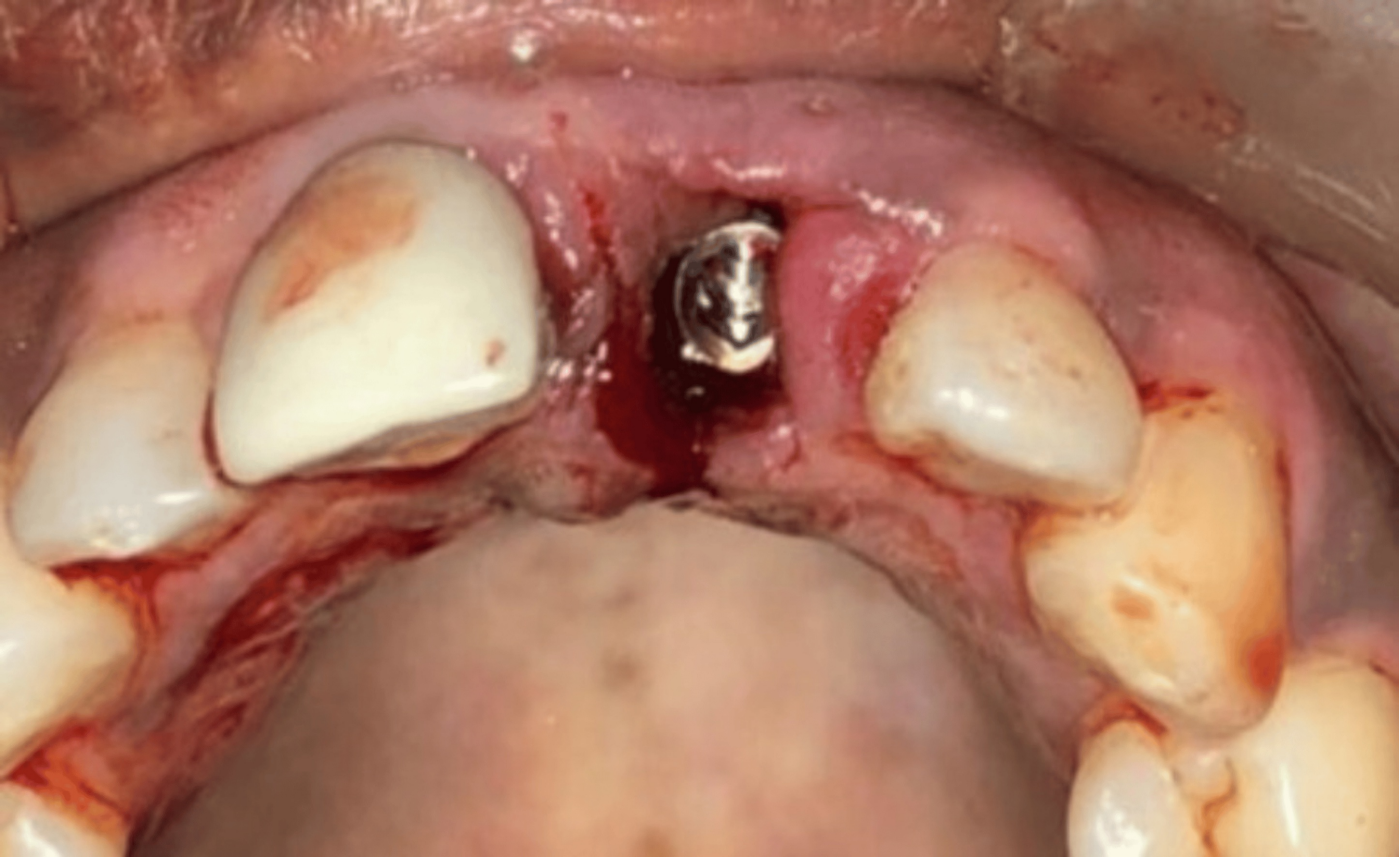
Immediate implant placed in region 21

**Figure 5:**
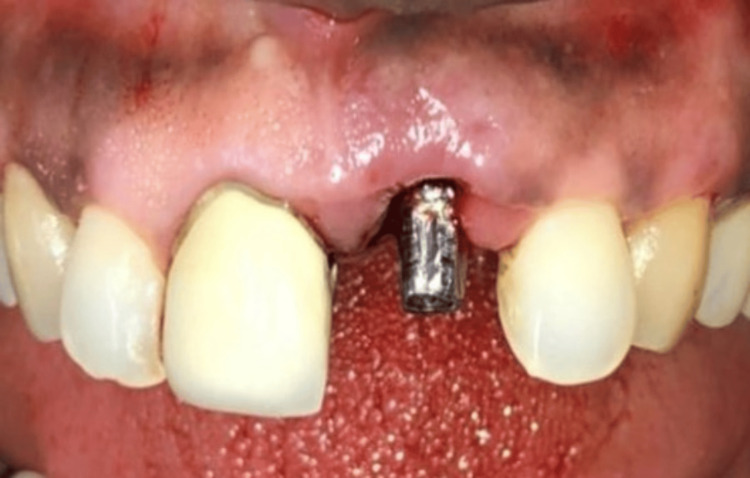
Prepared abutment screwed in region 21

**Figure 6:**
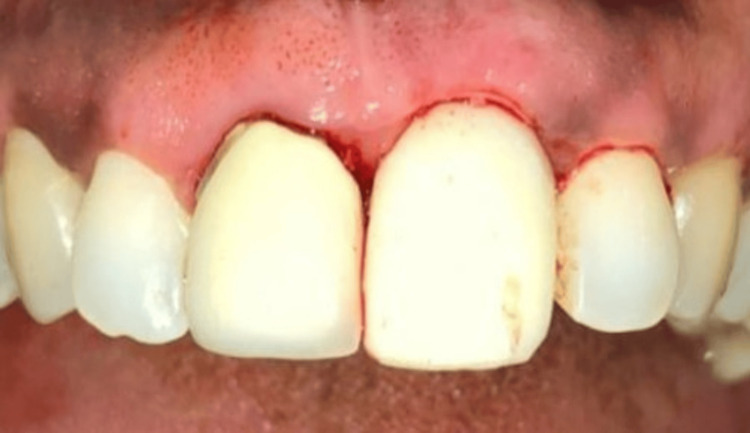
Temporary crown placed in region 21

Under the aseptic condition, local anesthesia was achieved by bilateral infraorbital nerve block and nasopalatine nerve block using 2% lignocaine hydrochloride with 1:80,000 adrenaline. The tooth was luxated using periotome and extracted without trauma, maintaining the soft tissue contour. Buccal bone intactness was checked. Socket length was measured and compared with CBCT findings. The prepared surgical stent was placed. The implant site was prepared by sequential drilling in the apical portion of the socket. The selected root form ADIN implant (ADIN Dental Implant Systems Ltd., Israel) of maximum length available (18 mm) was palatally placed in the socket, with a minimum 3 mm apical anchorage for implant stability, and 1 mm below the crestal bone level (Figure 4). In demanding cases, the nasal floor is engaged for apical anchorage. With orientation marking on the buccal aspect, the customised abutment was screwed into the implant (Figure 5). The prepared provisional crown was then fixed over the abutment, in infra-occlusion (Figure 6).

**Figure 7:**
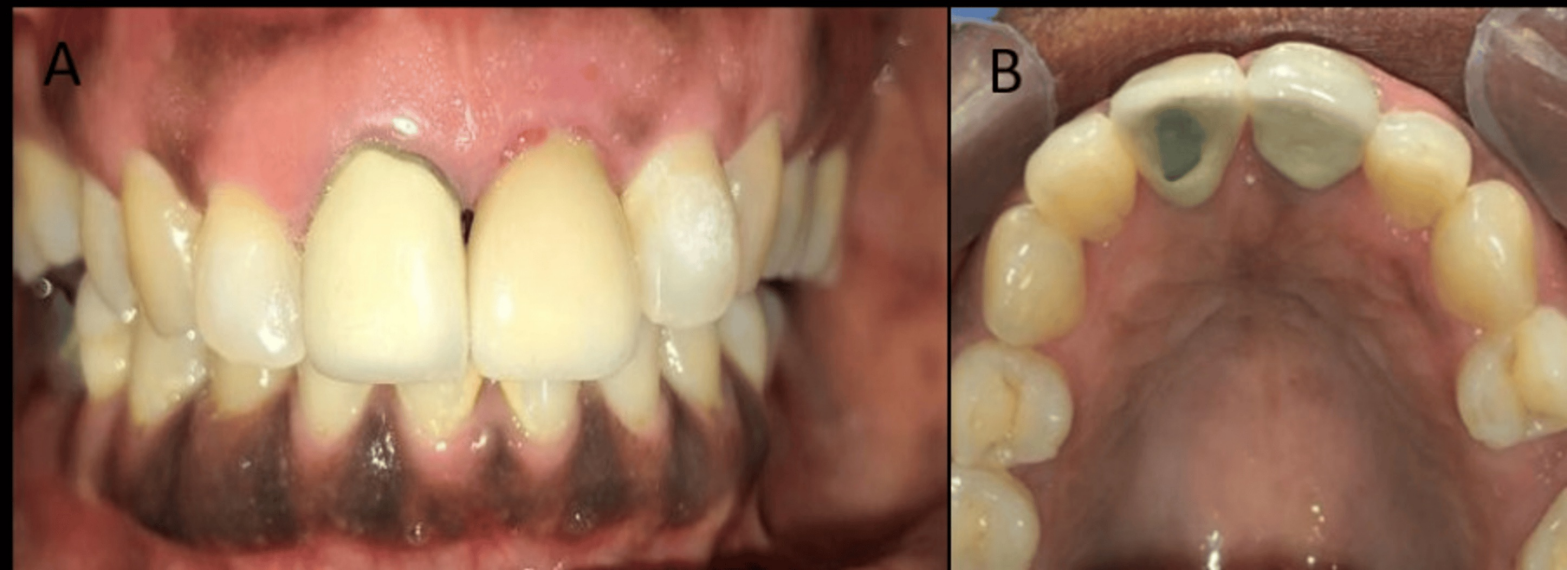
Final prosthesis – porcelain fused to metal crowns (PFM crown) in region 21 A: Frontal view; B: Palatal view

The provisional crown was trimmed and polished as required and splinted with light cure composite resin for stabilization. Coe-Pak was applied, postoperative evaluation was done, and the final prosthesis was placed after three months (Figure 7).

## Results

Functional provisional crowns were fabricated from the 3D RP model of the anterior maxillary region containing the prepared abutment. A total of 10 functional provisional crowns were immediately loaded on implants placed immediately following extraction in the anterior maxillary region in seven patients. Crestal bone loss (mesial and distal), thickness of buccal and palatal bone, and thickness of interdental papilla (mesial and distal) were evaluated for all patients.

IOPA were utilized to assess crestal bone levels on the mesial and distal aspects of the implants. A reference line was drawn from the cementoenamel junction (CEJ) of the adjacent teeth, and the distance from this line to the crestal bone level was measured. These measurements were taken at multiple time points, including immediately postoperatively (serving as the baseline), as well as at two, four, six, and eight weeks postop. Bone loss was quantified by calculating the difference in crestal bone levels compared to the baseline measurement. This approach allowed for tracking changes over time to assess the progression or stability of bone levels around the implant.

Statistical analysis of the data was done using IBM SPSS Statistics for Windows (version 26.0; IBM Corp., Armonk, NY). Descriptive statistics were calculated for the various clinical parameters, including mean, standard deviation, and minimum and maximum values. The normality of the data assessed using the Shapiro-Wilk test revealed that the data were not normally distributed. Therefore, further analysis was done using the non-parametric test. The level of significance in the present study was kept at p<0.05.

**Figure 8:**
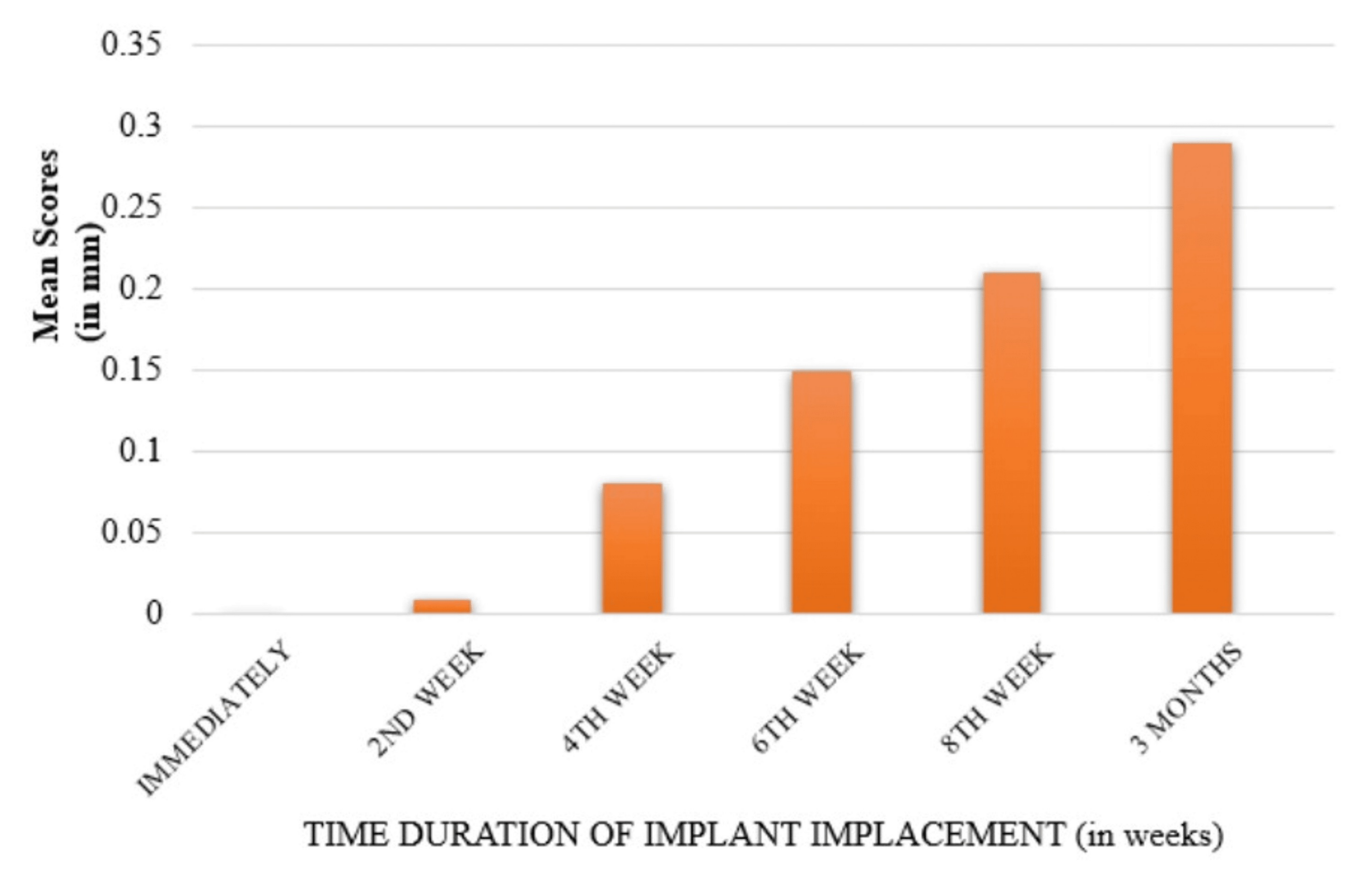
Comparison of crestal bone loss on the mesial side at different follow-ups

Crestal bone loss on the mesial side of the implants was evaluated using IOPA and assessed by an independent evaluator. At each follow-up interval - immediate postop, second week, fourth week, sixth week, eighth week, and third month - a reference line was drawn from the CEJ of the adjacent teeth to the crestal bone level at the implant site. The distance between this reference line and the crestal bone level was measured, providing values for bone loss over time. Mean values of crestal bone loss were recorded as 0 mm immediately postop, 0.009 ± 0.01 mm at two weeks, 0.083 ± 0.04 mm at four weeks, 0.15 ± 0.06 mm at six weeks, 0.21 ± 0.08 mm at eight weeks, and 0.29 ± 0.10 mm at three months. Statistical analysis revealed significant differences in bone loss across all follow-up periods. Pairwise comparisons showed significant variance between baseline and the sixth week, eighth week, and third month; between the second week and the eighth week and third month; and between the fourth week and the third month (Figure 8).

Crestal bone loss on the distal side was assessed and compared at various follow-up intervals using IOPA. The process involved measuring the distance from a reference line, drawn from the CE) of the adjacent teeth, to the crestal bone level at the implant site. The measurements were recorded at immediate postop, two weeks, four weeks, six weeks, eight weeks, and three months.

**Figure 9:**
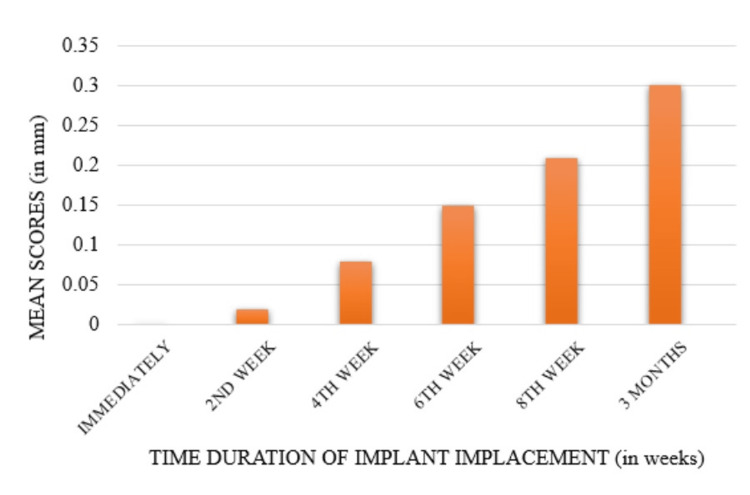
Comparison of crestal bone loss on the distal side at different follow-ups

The mean values of crestal bone loss on the distal side were 0 mm immediately postop, 0.02 ± 0.01 mm at two weeks, 0.08 ± 0.03 mm at four weeks, 0.15 ± 0.03 mm at six weeks, 0.21 ± 0.03 mm at eight weeks, and 0.30 ± 0.05 mm at three months. These values were calculated by measuring the vertical distance from the reference line to the crestal bone level at each time point and comparing these measurements over time. Statistical analysis indicated significant differences in bone loss between all follow-up periods, reflecting progressive bone loss around the implant (Figure 9).

Pairwise comparison between the groups shows a significant difference in the mean scores with statistical variance between as follows: baseline with sixth week, eighth week, and third month; second week with eighth week and third month; and fourth week with third month. The mean comparison between the mesial and distal sites of crestal bone loss at periodic levels shows statistically no significant difference (Table 1).

**Table 1:** Mean comparison of crestal bone loss of both mesial and distal sides at periodic follow-ups Mann-Whitney U test; p<0.05 considered statistically significant

Follow-ups	Mesial side		Distal side		P value
	Mean	SD	Mean	SD	
Immediately	0	0	0	0	-
2^nd^ week	0.009	0.01	0.02	0.01	0.13
4^th^ week	0.08	0.04	0.08	0.03	0.21
6^th^ week	0.15	0.06	0.15	0.03	0.17
8^th^ week	0.21	0.08	0.21	0.03	1.12
3 months	0.29	0.10	0.30	0.05	0.11

The bone width on the buccal side was evaluated and compared between preoperative and three-month postoperative values. The mean values are preoperatively 0.84 ± 0.3 mm and postoperatively 0.80 ± 0.2 mm. The p value is 0.73. The comparative mean values of the buccal bone width between preop and postop show no statistically significant difference among them.

The bone width on the palatal side was evaluated and compared between the preoperative and three-month postoperative values. The mean values are 1.90 ± 0.3 mm preoperatively and 1.82 ± 0.3 mm postoperatively. The p value is 0.16. The comparative mean values of palatal bone width between preop and postop show statistically no significant difference among them.

**Figure 10:**
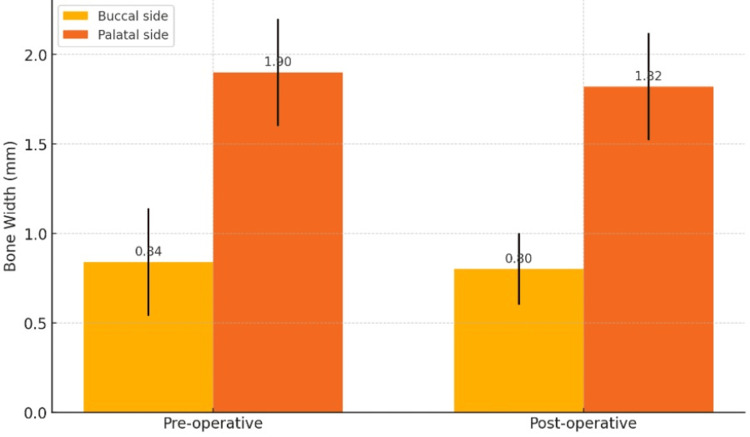
Comparison of preoperative and postoperative bone widths based on buccal and palatal sides

The mean comparison of the bone width between buccal and palatal aspects preoperatively shows a statistically significant difference, which indicates that the bone width mean scores of both buccal and palatal sites are not equivalent to each other. The mean comparison of the bone width between buccal and palatal aspects postoperatively shows no statistical significance among the interventions on both the sites, which indicates that the bone width mean scores of both buccal and palatal sites are equivalent to each other (Figure 10, Table 2).

**Table 2:** Mean comparison of the bone width (in mm) based on buccal and palatal sides *Mann-Whitney U test; p<0.05 considered statistically significant

Bone width	Buccal side (in mm)		Palatal side (in mm)		P value
	Mean	SD	Mean	SD	
Preoperative	0.84	0.3	1.90	0.3	0.04*
Postoperative	0.80	0.2	1.82	0.3	0.09

**Figure 11:**
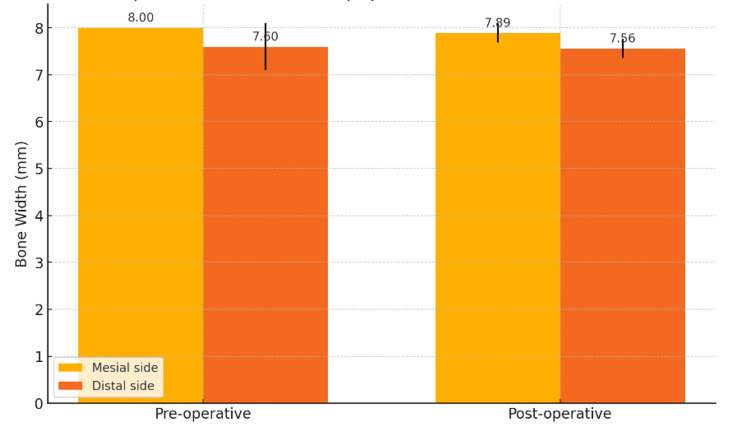
Comparison of preoperative and postoperative interdental papilla widths based on the mesial and distal sides

Study models were made at the preoperative and postoperative third month where the interdental papilla thickness was measured buccolingually on the mesial and distal aspects using a vernier caliper. The interdental papilla thickness at the preoperative and postoperative third month was evaluated and compared. The mean values are 8 ± 0 mm preoperatively and 7.89 ± 0.2 mm postoperatively. The p value is 0.21. The comparative mean values of the mesial interdental papilla width between preop and postop show statistically no significant difference among them. The interdental papilla thickness at preoperative and postoperative third months was evaluated and compared. The mean values are preoperatively (7.60 ± 0.5 mm) and postoperatively (7.56 ± 0.2 mm). The p-value is 0.13. The comparative mean values of the distal interdental papilla width between preop and postop show statistically no significant difference among them. The mean comparison of the interdental papilla width between the mesial and distal aspects at preoperative and postoperative shows no statistical significance among the interventions on both the sites, which indicates that the mean scores of the interdental papilla width between the mesial and distal aspects are equivalent to each other (Figure 11, Table 3).

**Table 3:** Mean comparison of interdental papillae based on the mesial and distal sides *Mann-Whitney U test; p<0.05 considered statistically significant

Bone width	Mesial side (in mm)		Distal side (in mm)		P value
	Mean	SD	Mean	SD	
Preoperative	8.0	0.0	7.60	0.5	1.000
Postoperative	7.89	0.2	7.56	0.2	0.82

**Figure 12:**
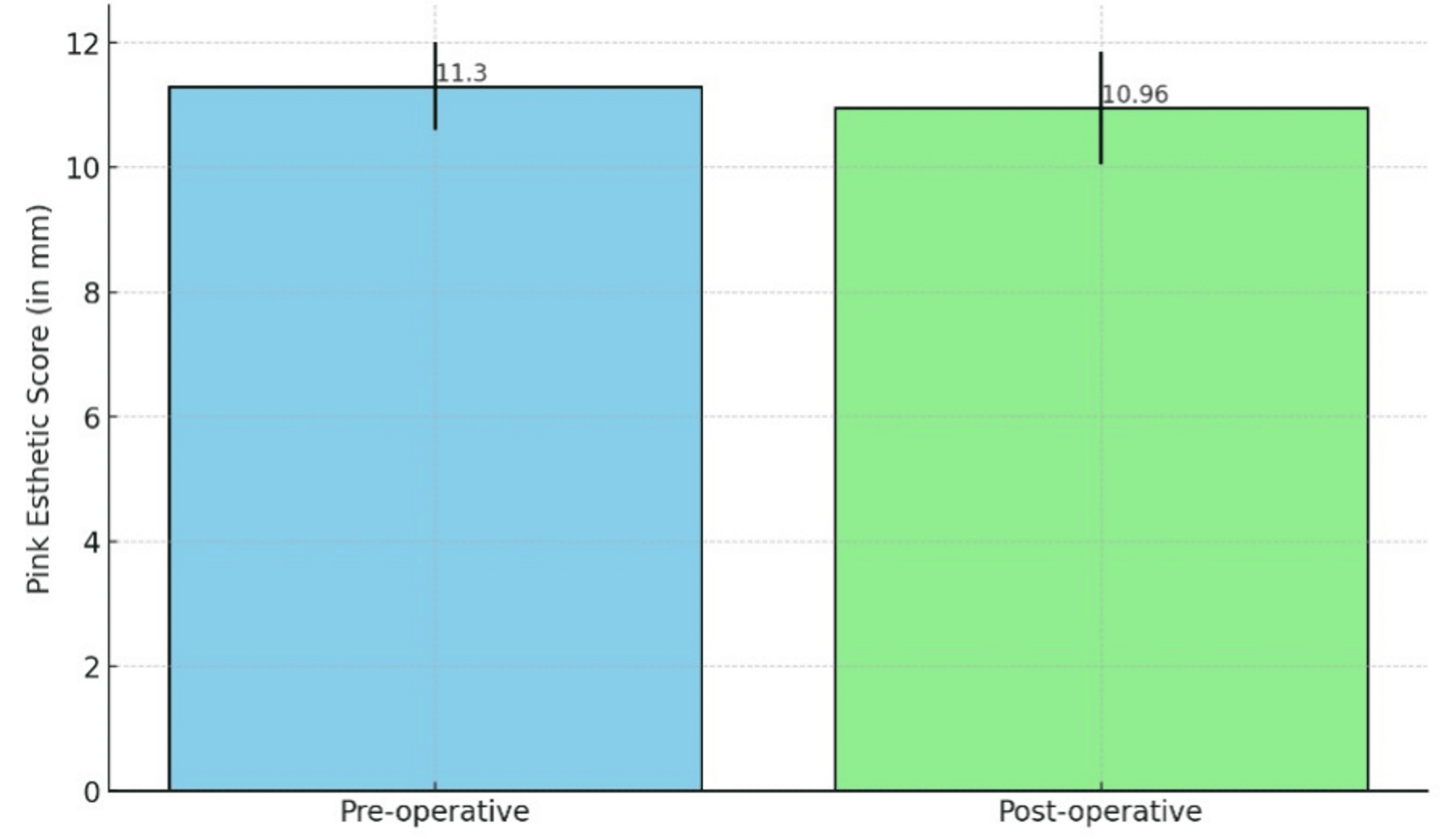
Comparison of the preoperative and postoperative pink esthetic scores (PESs)

The overall PESs at the preoperative and postoperative third month were evaluated and compared. The mean values are 11.30 ± 0.7 mm preoperatively and 10.96 ± 0.9 mm postoperatively. The p value is 0.18. The comparative mean values of the overall PESs between preop and postop show statistically no significant difference among them (Figure 12, Table 4).

**Table 4:** Mean comparison of the pink esthetic scores (PESs) *p<0.05 considered statistically significant using the Wilcoxon signed-rank test

PES	Mean	SD	Minimum value (in mm)	Maximum value (in mm)	P value
Preoperative	11.30	0.7	10	12	0.18
Postoperative	10.96	0.9	9	12	

## Discussion

Smile is a curve that makes our life straight. The aesthetics of a smile is associated with the harmonious appearance of the face. Thus, it has become increasingly important in the practice of modern restorative dentistry. In smile aesthetics, the appearance of the gingival tissues surrounding the teeth plays an important role. Along with the amount of gingival display, the contour also plays a major role in providing an optimal aesthetic appearance in the maxillary anterior region. Thus, the emergence profile that is maintained by the gingival contour is of utmost importance for the harmonious appearance of the smile. Therefore, any dental procedure performed in the maxillary anterior region will be an aesthetic challenge, and it should aim to preserve the gingival contour to maintain the emergence profile. This is not addressed by any conventional techniques.

Periodontium is the supporting structure for any tooth. Extraction of the tooth affects both the hard and soft tissue of the periodontium [1]. Dental implants have proven to be an efficient option for replacing teeth. As sequelae of extraction, the periodontium undergoes atrophy [1,2]. Qualitatively and quantitatively diminished periodontium affects the stability, function, and esthetics, which are to be provided by implant-supported restoration [3].

Tooth replacement in the maxillary anterior region should aim at preserving esthetics and function. Immediate placement of implants followed by atraumatic extraction of tooth in the maxillary anterior region became popular, which resulted in a significant reduction in post-extraction bone loss [4,5]. Alveolar socket walls are immediately supported by these root-form implants, thereby helping maintain their integrity and function [6]. The success of implant treatment should not only aim at survival rate and restoration but also should be free of complications with esthetic satisfaction [7]. Preserving the existing hard and soft tissues is an important factor for success in implant treatment. Atraumatic extraction is a technique that involves the removal of a tooth using specialized tools while causing minimal trauma to adjacent bone and soft tissue [8]. Periotomes and luxators can be used for atraumatic extraction.

In this study, periotomes were used to luxate the tooth/root structure and extracted using endodontic files. When adequate tooth structure is available, forceps are used for extraction after luxation.

Immediate placement of implants followed by atraumatic extraction of tooth in the maxillary anterior region became popular, which resulted in a significant reduction in post-extraction bone loss [9]. However, there are soft tissue changes such as papillary recession, resulting in the formation of a black triangle after fixing the prosthesis, loss of the width of the attached gingiva, alteration of gingival contour affecting the emergence profile during final prosthesis placement, and loss of mucosal attachment around the implant surface leading to periimplantitis [10].

For a tooth in the maxillary anterior region, the emergence profile is of utmost importance for esthetics. The soft tissue contour and anatomy are lost, as the flap is advanced and sutured, resulting in loss of the emergence profile [11,12]. In order to maintain the bone architecture along with the soft tissue profile in the maxillary esthetic zone, immediate provisional prosthetic restoration on immediately placed implants following atraumatic extraction will be of better choice. It can be done as a single-stage technique avoiding the need for placing healing abutment [13,14].

"Tooth in a day" has become huge a interest among clinicians, as well as patients by immediate replacement of teeth that cannot be saved in the maxillary esthetic zone with an implant-supported restoration. Many studies reported good success rate of this procedure [15]. In our study, immediate loading on immediately placed implants following atraumatic extraction in the maxillary anterior esthetic region was done to avoid bone loss and to preserve the soft tissue contour, thereby maintaining the emergence profile of the final prosthesis. A total of 10 immediately loaded implants placed in seven patients were included in this study.

Emergence profile is the contour of a tooth or restoration, such as a crown on the natural tooth or dental implant abutment, as it relates to the adjacent tissues (GPT-8). In simple terms, it is the contour of the tooth as it emerges from the gingiva [15]. In the case of dental implants, the emergence profile is the contour of restoration as it emerges from the implant platform.

In many studies, abutment preparation was done directly in the patient’s mouth. This preparation needs trimming of the abutment, which may affect the primary implant stability, as it produces vibration. The crown can be fabricated in many ways such as an impression made and transported to the laboratory where the provisional crown is fabricated; a crown prepared with composite materials directly in the patient’s mouth using shell crowns; or fabricated in the chairside with prepared dental cast. The fabricated functional provisional crown is transferred to the patient’s mouth where it is trimmed according to requirements. This will increase the operating time, leading to the need for administration of more local anesthetic dosage unnecessarily. Crown trimming also produces vibration to the implant, which again may affect the stability of immediately placed implants [16]. To avoid these complications, in our study 3D RP model of the anterior maxillary region is used for abutment preparation and crown fabrication.

The preparation of 3D RP models of the maxilla and mandible is as follows. DICOM data of the patient’s CBCT/CT scan were obtained. Using “Mimics” software, a simulation of the extracted socket was done by deleting the tooth portion in the virtual model. 3D CAD models of the maxilla with the simulated extracted socket and mandible were created, which were converted to binary STL file formats. By the additive manufacturing technique, 3D RP models are printed from the STL file (Primaeam Solutions Private Limited, Chennai, India).

In the 3D RP model of the maxilla without the tooth of interest (simulated extraction socket), drilling was done in the apical portion of the simulated extracted socket. We here selected the maximum length of the implant available (18 mm), which is placed in the simulated socket 1 mm below the marginal bone level. Implant anchorage was achieved by placing at least 3 mm beyond the apex of the socket. Through sagittal sections of the CBCT/CT scan, the socket position of either buccal/middle/palatal is known. In 3D RP models, angulation of implant placement is more palatal, and it also depends on the position of the socket. This aids in obtaining screw-retained final prosthesis, which is preferred to cemented restorations.

We have selected the implant width size as 1 mm less than that of the socket width. This is to maintain the jumping distance, which may or may not require grafting, as good healing occurs irrespective of grafting. It also avoids engaging the buccal cortex, thereby maintaining the buccal bone thickness. In the implant, the stock abutment is screwed and prepared according to the amount of interocclusal clearance available using a 3D RP model of the mandibular anterior region. Orientation marking is done on the prepared abutment for accurate transfer to the patient. The impression of the 3D model with the prepared abutment is made. The functional provisional crown is fabricated in the prepared dental cast/3D model, according to the adjacent and opposing teeth.

The 3D RP models that were printed constitute only the hard tissue portion of the anterior maxilla. Soft tissues were not included in the models. Hence, the crowns that are fabricated out of these 3D RP models require more trimming, compared to those fabricated out of plaster casts that are made from direct mouth impressions.

Clinically, in the maxillary esthetic region, following atraumatic extraction of the tooth, implants were placed immediately and loaded with a functional provisional crown prepared pre-operatively in the 3D-printed RP model of the anterior maxillary region [17]. The functional provisional crowns were trimmed and adjusted sufficiently in order to avoid contact with opposing teeth and to provide relief, allowing interdental papilla growth. Sometimes, this trimming has compromised the crown esthetics to some extent, but, still, the soft tissues are preserved as desired. For patients with deep bites, it is difficult to fabricate and load provisional crowns immediately.

The problem that was faced during the study is the loosening of the provisional crowns. By the time the patient arrives hospital to refix the crown, epithelization of the site has occurred. The incision was required to expose the screwed customized abutment, and the functional provisional crown is refixed. These provisional crowns are initially fixed in the interdental area using light cure composite resin. Due to loss of retention, splinting to adjacent teeth was done in our study. Menawhile, in some studies [6], though they have observed loss of retention of provisional crowns frequently, they have not advised any solution.

Thus, compared to previous studies, immediate loading of immediately placed implants through our method has the benefits of not only maintaining the soft tissue contour with emergence profile, but also it maintenans primary implant stability, has less chairside time, has short duration of surgery, and can avoid second surgery for the placement of a healing abutment [16,17].

In the 10 cases included in our study, one case presented with pyogenic granuloma. The pathology was treated successfully and later proceeded with immediate implant, followed by the immediate loading treatment protocol. Pathology did not affect the outcome of the treatment. Bone and soft tissue status was assessed postoperatively. Crestal bone loss, buccal and palatal bone thickness, interdental papilla thickness, and PESs were assessed in our present study.

Limitations: This study on immediate implant placement and loading in the maxillary anterior region had several limitations. The small sample size and absence of a control group limit the generalizability and comparison with other methods. The short-term follow-up does not provide long-term success insights. The 3D RP models used lacked soft tissues, necessitating additional crown trimming, affecting fit and esthetics. Issues with loosening provisional crowns required further interventions, and deep bite patients posed challenges. The technical complexity of using 3D RP models limits its applicability in less equipped settings. Balancing function and esthetics often led to compromises, and retention issues needed splinting to adjacent teeth.

## Conclusions

The concept of immediate functional loading on immediately placed implants following extraction helped maintain the original soft tissue contour by acting as a template during the healing process. Thus, it is important to provide immediate prosthetic provisional restoration. This technique has resulted in a significant reduction of marginal bone loss and soft tissue alteration, thereby preserving alveolar bone height and width, interdental papilla, and marginal gingiva contour in the post-extraction phase, compared to previously mentioned other techniques.

Thus, immediate loading of functional crowns in the anterior maxillary region following immediate placement of dental implants in the extraction socket will maintain the status of both bone and surrounding soft tissues, which is certainly an esthetic and functional benefit to the patient, by avoiding wearing uncomfortable removable dentures during the healing period, and reduces the overall time and number of operating sessions. Appropriate case selection, proper diagnosis, and treatment planning are important to obtain successful outcomes. Though soft tissue outcomes in our study have improved compared to previous studies, we had some limitations as the 3D RP model did not contain the soft tissue counterpart. Future studies can be done using models, including soft tissues for better results.
